# Septic gonococcal arthritis in a pediatric patient: Rare case report

**DOI:** 10.1016/j.ijscr.2021.105701

**Published:** 2021-02-24

**Authors:** Atul Saini, Clayton Eichenseer, Andrew Meyers, Petros Frousiakis

**Affiliations:** a Graduate Medical Education, Orthopaedic Surgery Residency Program, Community Memorial Health System, Ventura, CA, USA; b Community Memorial Health System, Ventura, CA, USA

**Keywords:** Gonococcal, Arthritis, Septic, Case report, Pediatric

## Abstract

**Introduction:**

Septic arthritis is an orthopedic emergency that requires rapid diagnosis and treatment due to the rapid destruction to cartilage. The responsible organism and etiology differs depending on patient age, especially in children. Gonococcal Arthritis in toddlers is a rare occurrence with few documented cases in the literature. An orthopedic surgeon is likely not to come across this either in training or through their careers. Consequently, its presentation and subsequent treatment algorithms leave several gray areas.

**Presentation of case:**

In this case report, we present a rare and not so straightforward presentation of a toddler with septic gonococcal arthritis along with a summary of treatment considerations described in the current literature and the course of treatment for this patient.

Our patient is a toddler who originally presented to the emergency department with shoulder and knee pain for several days after an unwitnessed fall. He was subsequently discharged and presented again the next day with a knee effusion and elevated inflammatory markers. An MRI showed a large joint effusion without any underlying abscess or osteomyelitis to explain his elevated inflammatory markers. A knee aspiration was subsequently preformed which eventually grew out *Neisseria Gonorrhea* on hospital day 3 after the patient had been on antibiotics. He was taken back for an arthroscopic irrigation and debridement for definitive treatment.

**Clinical discussion/conclusion:**

Disseminated gonococcal infection in toddlers is a rare occurrence without much information in the literature and should not be dismissed as a differential. We recommend a high index of suspicion with thorough work up. We also recommend surgical management of a septic joint due to DGI diagnosed via arthrocentesis (gross purulence, symptoms not improving on medical therapy, positive aspiration cultures, elevated synovial cell counts, and medically unstable patients) given the sequelae of medical management alone. The importance of interdisciplinary team collaboration that include pediatrician, infectious disease specialist, social worker, and government child safety associations is pivotal.

## Highlights

• *Neisseria Gonorrhea* is one of the most common sexually transmitted diseases.
• Even rarer is septic gonococcal arthritis in the pediatric population.
• The medical treatment of gonococcal arthritis differs from treating any other infectious arthritis.
• A case can be made for operative intervention for patients with septic gonococcal arthritis.

## 1 Introduction

*Neisseria Gonorrhea* (*N. Gonorrhea*) is one of the most common sexually transmitted diseases. In 2017, 555,608 cases of gonorrhea were reported in the USA [1]. Its risk factors include unsafe sexual practices and multiple partners through oral, anal, or vaginal contact [2,3]. It was originally identified by Albert Niesser in 1879 [4]. *Neisseria Gonorrhea* is a gram-negative intracellular diplococci, growing readily on thayer-martin agar in labs. It may be symptomatic or asymptomatic, leading to greater spread. Though it can affect any age group, the classic presentation is usually seen in young adults [5]. Rarely does it present in children less than the age of 10 [6].

*Neisseria Gonorrhea* either can manifest as a localized disease process or disseminated gonococcal infection (DGI). It is estimated that only 0.4–3% of all gonococcal infections develop into DGIs [7]. Gonococcal arthritis is often an accompanying symptom of disseminated gonococcal infection. It can be either monoarticular (40% of the time) or a combination of tenosynovitis, polyarthralgia or migratory polyarthritis, and skin lesions (60% of the time) [8].

The treatment for *Neisseria Gonorrhea* is medical management in the form of antibiotics, usually a combination of ceftriaxone and azithromycin, contingent on there being no resistance. This is the case for the infection in both its local form and disseminated form [3]. The medical treatment of gonococcal arthritis differs from treating any other infectious arthritis, which entails irrigation and debridement followed by intravenous antibiotics as the standard of care. However, there are case reports in the literature that point to sequelae of medical management alone of gonococcal arthritis, including septicemia, chronic osteomyelitis, unremitting pain, and functional deficits [9,10]. We present a rare, unique, and challenging case of gonococcal arthritis in a toddler, along with current treatment recommendations, an area where the literature it is not deficient, but ambiguous as well in terms of treatment algorithm. Of note, this case presentation has been designed in-line with the Surgical Case Report (SCARE) 2020 Guidelines [25].

## 2 Case presentation

**Fig. 1:**
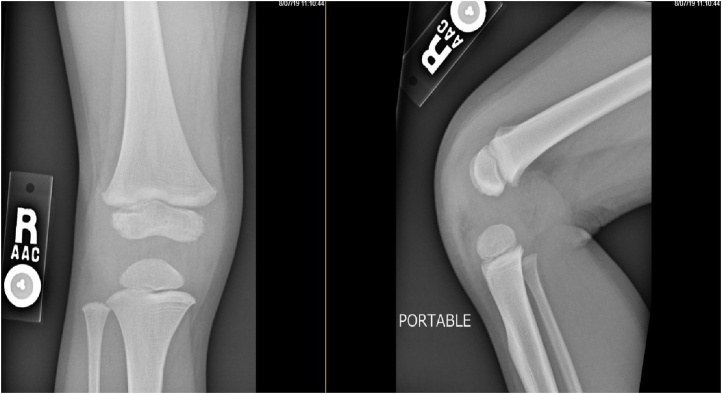
Radiographs of the right knee taken upon the patient's initial evaluation to the emergency department. No osseous or soft tissue abnormalities are noted.

This case is a toddler with up to date vaccinations and no significant past medical history. The patient initially presented in the Emergency Department (ED) at our community hospital after having persistent left shoulder pain and right ankle pain. The mother noted the symptoms approximately three days prior to presentation to the ED. The mother also reported that the patient had a worsening right lower extremity limp, and the patient had a recent fall from a high bed. The treating ED clinician examined the patient and ordered radiographic imaging of the left shoulder, right ankle, and right knee [Fig. 1]. Imaging did not demonstrate any pathology, and the patient was discharged with instructions to follow up with an orthopedic surgeon as an outpatient.

Past Surgical History: Denies

Allergies: Denies

Family History: No known illnesses within the family

Social History: Denies any alcohol, smoking, recreational drug use. Lives with mother and father along with sibling.

Review of Systems: 10 point review of systems negative unless otherwise noted in below

**Fig. 2:**
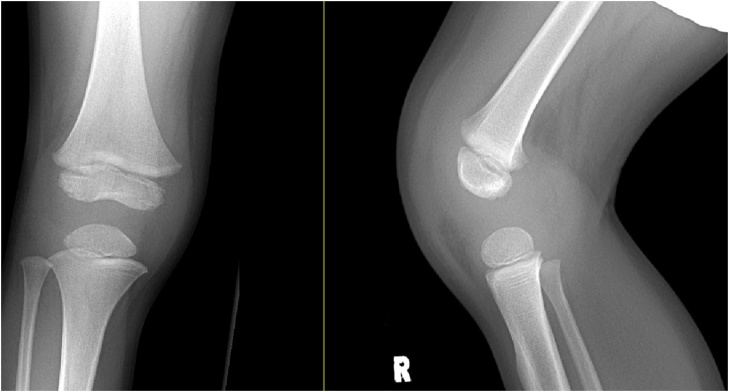
Radiographs taken of the right knee the following day upon re-evaluation. An effusion can be appreciated along with soft tissue swelling without apparent osseous involvement.

**Fig. 3:**
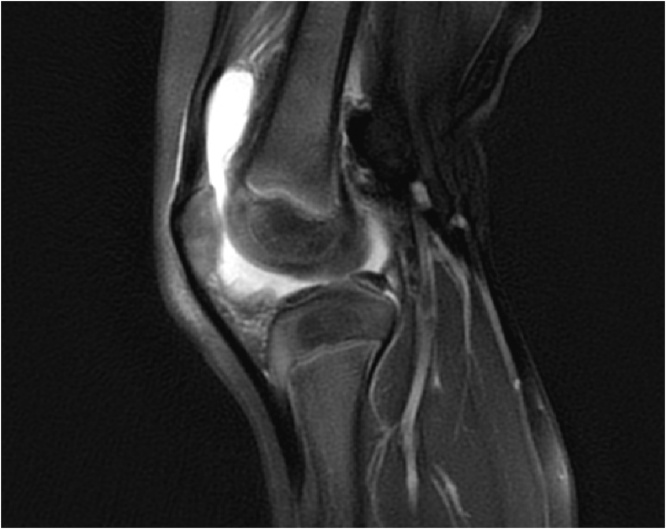
MRI right knee (Sagittal T2 Fat Suppression sequence): Large Knee Effusion with no abnormal bone marrow signal or fluid collection to suggest abscess.

The evening after discharge from the ED, the patient's mother further inspected the patient’s knee, which was found to be swollen and warm to the touch. The mother reported that the patient had 103 degrees Fahrenheit temperature. The patient was unable to bear any weight on the right lower extremity. The patient was brought back to the ED. Both pediatrics and orthopedic services were consulted. At presentation, the patient was found to be afebrile but had received children's Tylenol at home just before arrival. Labs were drawn (WBC 11.9, CRP 12.8 mg/dl, and ESR 54), and a physical exam was performed and radiographs repeated [Fig. 2]. A visible and palpable effusion was present. The patient could actively range the knee from 10 degrees to 90 degrees of flexion, acknowledging the painful range of motion. It was recommended by the orthopedic team to obtain an MRI for further evaluation. The MRI demonstrated a large joint effusion with no evidence for osteomyelitis, abscess, or other notable pathology [Fig. 3]. The orthopedic service determined the patient warranted a knee aspiration, which resulted in 27,400 white blood cells and 71% polymorphonuclear leukocytes on cell count, no organisms on gram stain, and no crystals upon evaluation. The patient was admitted to the pediatric unit for observation, while cultures were pending. Vancomycin was started in the emergency department as well.

Overnight, the patient had a temperature of 101.6 degrees Fahrenheit, but the fever was controlled with IV Toradol at a 6-h interval. In the morning, the patient's knee pain had minimal improvement, and left shoulder pain had fully resolved. The highest on the differential included transient synovitis. Thus, the patient was maintained on IV Toradol and kept on close observation. On hospital day 2, the patient's symptoms showed significant improvement. The Toradol was then discontinued. Cultures had shown no growth. CRP was redrawn, and the result showed a decline to 7.4 mg/dl from 12.8 mg/dl. Although the patient was showing improvement, there was still a deep concern for infection. At this point, vancomycin was discontinued, and the patient was transitioned to oral Bactrim and Cefdinir per the pediatric team for coverage of both methicillin resistant staphylococcus aureus (MRSA) and *Kingella Kingae*. The patient was held one more night to monitor for any recurrence of or worsening of symptoms. CRP was repeated in the morning, now 4.6 mg/dl, and the patient's symptoms continued to improve.

As the patient was being prepared for discharge on hospital day three, the microbiology lab reported that the cultures taken from the initial aspiration had resulted in the rare growth of N. Gonorrhoeae. Because of this new positive culture, although an unusual presentation of septic arthritis, it was recommended by the orthopedic service that the patient undergo arthroscopic irrigation and debridement of the right knee. The antibiotic regimen was then again tailored to cover *N. Gonorrhea* with ceftriaxone. At this point, it was determined to consult social work due to the patient’s age and association of *N. Gonorrhea* with sexual transmission. After receiving the report of positive culture, the patient was taken to the operating room for right knee arthroscopic irrigation and debridement with the attending surgeon, Dr. Frousiakis.

**Fig. 4:**
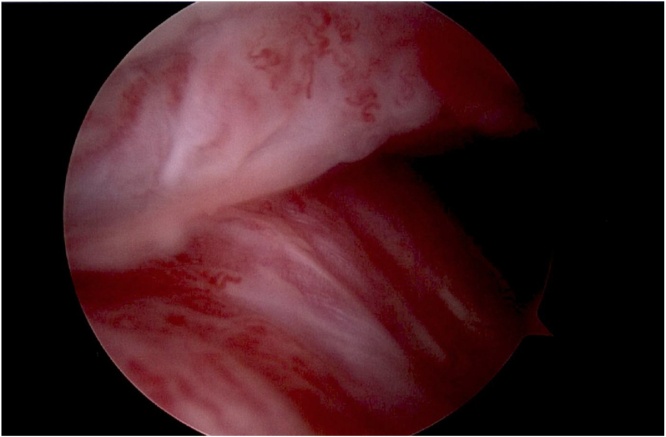
Intra-operative picture during knee arthroscopy demonstrating hypertrophic and inflamed synovitis in the supra-patellar pouch.

The patient was prepped and draped in a normal sterile fashion. Time out was performed; a marking pen was then used to mark out the preplanned anteromedial and anterolateral portal incisions. The anterolateral cannula was first introduced, and purulent fluid was observed evacuating the joint. This fluid was captured in a sterile container for further analysis. A 2.7 mm scope was then inserted. After the anteromedial portal was created, six liters of normal saline were used to irrigate the knee, and a standard diagnostic arthroscopy was performed [Fig. 4]. No synovium was debrided or sampled. The patient tolerated the procedure well with no complications.

On hospital day four, the social worker contacted Child Protective Services for suspicion of sexual abuse. A tertiary children's hospital was contacted to determine the best antibiotic regimen. Their recommendation was a seven day course of ceftriaxone. Placing a peripherally inserted central catheter was under consideration. However, because of the ongoing Child Protective Services investigation, it was determined that the patient should remain admitted for the full duration of therapy as further information was collected, and STD testing was performed. On hospital day eight, post-operative day four, CRP was drawn, which showed a decline at 0.8 mg/dl from 2.4 mg/dl. The patient continued to progress appropriately with no residual knee pain and ambulated with full weight-bearing. The patient was discharged on hospital day ten with instructions to follow up with an orthopedic surgeon, pediatrician, and infectious disease expert locally where the family resides.

## 3 Discussion

This paper aims to detail the case of DGI presenting as septic arthritis in a toddler, a disease rarely documented in this subgroup of the population. The gonorrhea infection rate is highest in adolescents and young adults according to the CDC, which does not report an infection rate for individuals less than ten years old, underscoring the rarity of disease prevalence in this age group [1]. Of those infected with *Neisseria gonorrhea*, only 0.5%–3% develop DGI [11].Gonococcal arthritis is a subgroup of DGI. The initial gonorrhea infection is most commonly acquired via sexual contact, including genital, anorectal, or oral contact with an already infected individual. Furthermore, patients are frequently asymptomatic at the time of initial inoculation, with more than 50% of rectal infections and up to 90% of pharyngeal infections not showing any signs or symptoms. This asymptomatic period allows the bacteria to disseminate hematogenously and seed joints until declaring itself as an infection. The bacteria can seed multiple joints owing to the well-documented migratory polyarthritis with a predilection for the knees, paralleling our patient’s presentation of left shoulder, right ankle, and eventual right knee pain. Additionally, blood cultures are negative in over two-thirds of cases of purulent gonococcal arthritis [12]. Our patient’s synovial cultures from the aspiration yielded a positive result on hospital day 3, contributing to a delay in targeted antibiotic therapy and further establishing the importance of keeping DGI high on the differential when patients of all ages present with symptoms of migratory polyarthritis. History and physical exam are important for many reasons, especially in the pediatric population who cannot always provide a history or describe their symptoms, as was the case with our patient.

Gonococcal arthritis comprises two clinical forms, including arthritis-dermatitis syndrome and localized septic arthritis, with some patients exhibiting characteristics of each type simultaneously. The typical triad of arthritis–dermatitis syndrome consists of tenosynovitis, dermatitis, and polyarthralgia, frequently associated with constitutional symptoms. Localized septic arthritis typically presents as a monoarthritis or asymmetric oligo-or polyarthritis with pain and swelling of one or more joints, most commonly the knees, ankles, wrist, and elbow [13]. Our patient presented with characteristics of each. However, their presentation was more consistent with the ladder given their left shoulder, right ankle, right knee pain, and lack of dermatitis.

The appropriate evaluation of a patient presenting with joint swelling and effusion includes arthrocentesis with synovial fluid analysis, which will typically demonstrate a white blood cell (WBC) count of greater than 50,000 cells/mm^3^ in the setting of septic arthritis. Occasionally, a non-diagnostic WBC count of fewer than 10,000 cells/mm^3^ is found due to reduced glucose and elevated LDH, necessitating additional evaluation. Furthermore, the bacteria in patients presenting with localized purulent *N. gonorrhea* arthritis will only be isolated in approximately 50% of synovial fluid specimens and even less reliably in the arthritis-dermatitis form. Nucleic acid amplification testing (NAAT) has greater than 75% sensitivity for synovial fluid analysis compared to culture and should be performed when available [13,14].

When a child beyond the newborn phase tests positive for *N. Gonorrhea*, sexual abuse must be considered as a possible source [2,15]. Family, friends, and others involved in the patient’s life must be questioned, as obtaining an accurate history, can be difficult for the pediatric patient, consistent with our case example [16,17]. Assessing family dynamics and potential red-flag answers to questioning, such as inconsistent stories, can help the provider elucidate whether or not sexual abuse has occurred. Evaluation should consist of a thorough physical exam with genital, pharyngeal, and rectal cultures [18]. Additionally, these patients should be tested for other STIs, including Chlamydia, Syphilis, Hepatitis B, and HIV [19]. Gonococcal infections in the pediatric population must be reported to public health authorities and child protective services [20]

Treatment of gonococcal infections varies based on the type of infection, age, and weight of the patient. Geographical resistance patterns must also be considered. For prepubertal individuals with DGI weighing less than 100 lb (45 kg), Ceftriaxone 50 mg/kg/day (maximum 1 g/day) intravenously or intramuscularly once per day for seven days is appropriate. Patients treated with this protocol typically do not require follow-up cultures [11].

While medical management is the mainstay of treatment for DGI, it is important to consider surgical decompression. There are several devastating sequelae of medical management alone, most importantly, osteomyelitis despite appropriate antibiotic treatment [21,22]. We recommend aspiration of the affected joint for cell count and cultures. Aspiration of frank pus should be treated with arthroscopic versus open incision and drainage to prevent chondrolysis in the face of enzymatic degradation and future post-infectious arthritic changes [23,24]. We opted for arthroscopic treatment to limit morbidity from a larger procedure. There is no literature demonstrating that gonococcal arthritis will have different cell count values that should prompt operative treatment. Therefore, we recommend a cell count cut off 50,000 WBC or PMNs greater than 85% to prompt operative intervention. A case can be made for operative intervention for patients not improving with medical management or decompensation despite medical management.

Unfortunately, this patient followed with a different provider after discharge which precluded clinical and radiographic follow up both short term and long term, highlighting a limitation of our case report.

## 4 Conclusion

Disseminated gonococcal infection in toddlers is a rare occurrence without much information in the literature and should not be dismissed as a differential. We recommend a high index of suspicion with thorough work up. We also recommend surgical management of a septic joint due to DGI diagnosed via arthrocentesis (gross purulence, symptoms not improving on medical therapy, positive aspiration cultures, elevated synovial cell counts, and medically unstable patients) given the sequelae of medical management alone. The importance of interdisciplinary team collaboration that include pediatrician, infectious disease specialist, social worker, and government child safety associations is pivotal.

## Declaration of Competing Interest

The authors declare that there is no conflict of interests regarding the publication of this paper.

## Funding

The only source of funding utilized was the Community Memorial Health System: Graduate Medical Education program. The funding was utilized to submit this manuscript. There was otherwise not outside funding nor sponsorship used.

## Ethical approval

The Community Memorial Health System IRB exempted our study due to its nature of being a case report with the understanding that patient’s parent would provide written consent to the study. If there are any questions or concerns regarding the ethics of this patient, please contact Dr. Graal Diaz at gdiaz.con@cmhshealth.org.

## Consent

Written informed consent was obtained from the patient’s parent for publication of this case report and accompanying images. A copy of the written consent is available for review by the Editor-in-Chief of this journal on request.

## Author contribution

All authors contributed equally to the composition of this manuscript.

## Registration of research studies

Not applicable.

## Guarantor

Atul Saini, DO

## Provenance and peer review

Not commissioned, externally peer-reviewed.
